# Enhanced Dupuytren's disease fibroblast populated collagen lattice contraction is independent of endogenous active TGF-β_2_

**DOI:** 10.1186/1471-2474-5-41

**Published:** 2004-11-12

**Authors:** Raymond Tse, Jeffrey Howard, Yan Wu, Bing Siang Gan

**Affiliations:** 1 Department of Surgery, The University of Western Ontario, London, Ontario, Canada; 2 Department of Biochemistry, The University of Western Ontario, London, Ontario, Canada; 3 Department of Physiology and Pharmacology, The University of Western Ontario, London, Ontario, Canada; 4 Cell & Molecular Biology Laboratory, Hand and Upper Limb Centre, Lawson Health Research Institute, St. Joseph's Health Centre, London, Ontario, Canada

## Abstract

**Background:**

Dupuytren's disease (DD) is a debilitating fibro-proliferative disorder of the hand characterized by the appearance of fibrotic lesions (nodules and cords) leading to flexion contractures of the fingers and loss of hand function. Although the molecular mechanism of DD is unknown, it has been suggested that transforming growth factor-β_2 _(TGF-β_2_) may play an important role in the underlying patho-physiology of the disease. The purpose of this study was to further explore this hypothesis by examining the effects of TGF-β_2 _on primary cell cultures derived from patient-matched disease and normal palmar fascia tissue using a three-dimensional collagen contraction assay.

**Methods:**

Fibroblast-populated collagen lattice (FPCL) contraction assays using primary cell cultures derived from diseased and control fascia of the same DD patients were studied in response to exogenous TGF-β_2 _and neutralizing anti-TGF-β_2 _antibodies.

**Results:**

Contraction of the FPCLs occurred significantly faster and to a greater extent in disease cells compared to control cells. The addition of TGF-β_2 _enhanced the rate and degree of collagen contraction in a dose-dependent fashion for both control and diseased cells. Neutralizing anti-TGF-β_2 _antibodies abolished exogenous TGF-β_2 _stimulated collagen contraction, but did not inhibit the enhanced basal collagen contraction activity of disease FPCL cultures.

**Conclusions:**

Although exogenous TGF-β_2 _stimulated both disease and control FPCL contraction, neutralizing anti-TGF-β_2 _antibodies did not affect the elevated basal collagen contraction activity of disease FPCLs, suggesting that the differences in the collagen contraction activity of control and disease FPCL cultures are not due to differences in the levels of endogenous TGF-β_2 _activity.

## Background

Dupuytren's disease (DD) is a fibro-proliferative disorder of the palmar fascia (PF) characterized by the formation of fibrous nodules and cords [1]. The disease results in digital contractures, leading to loss of hand function. Surgical excision of the diseased PF is currently the principal form of management since the lack of a clear etiology has precluded the development of other effective and rational forms of treatment.

Since Baron Guillaume Dupuytren's classical description of the disease in 1831, multiple clinical associations have been described, however, no clear molecular mechanism for the disease has been established [2]. Histochemical studies of DD have demonstrated the presence of myofibroblasts [3], increased production of type III collagen [4-7], and alterations in other extra-cellular matrix proteins including various fibronectin isoforms [8-14]. These biological features are characteristic of abnormal growth factor regulation, specifically fibrogenic cytokines such as transforming growth factor-beta (TGF-β). Several studies have documented TGF-β expression in DD palmar fascia using RT-PCR [15], *in-situ *hybridization [16], and immunohistochemistry [16-18], while others have shown that TGF-β can stimulate cell proliferation [18-20] and promote myofibroblast differentiation *in vitro *[21]. As a result of these and other studies it has been suggested that an aberrant TGF-β activity may be involved in the pathogenesis of DD.

In this study, we chose to focus on TGF-β_2 _and its effects on collagen contraction *in vitro *using a three-dimensional fibroblast populated collagen lattice (FPCL) contraction assay and DD patient-matched disease and control primary cell cultures. Previous reports have examined the role of TGF-β in DD by comparing disease fibroblasts to 'control' fibroblasts obtained from transverse carpal ligament material obtained from patients undergoing carpal tunnel release (CTR). By contrast, the control fibroblast cultures used in this study were established from unaffected PF tissue from the same patient, thus providing us with unique patient- and tissue- matched control cultures. The observed phenotypic differences between patient/tissue-matched control and disease FPCL cultures, specifically elevated collagen contraction activity, and β-catenin and fibronectin (Fn) expression in disease cells [22-24], raises the intriguing possibility that pro-fibrotic factors, such as TGF-β_2_, may be regulating these disease-associated events *in vitro*, since TGF-βs are known to promote fibroblast mediated collagen contraction [21,25,26] and up-regulate collagen, Fn and β-catenin [20,27-30]. As described in detail below, we have found that exogenous TGF-β_2 _could significantly stimulate 'normal' and disease FPCL contraction in a dose-dependent manner. While neutralizing anti-TGF-β_2 _antibodies completely blocked exogenous TGF-β_2 _stimulated FPCL contraction they had no effect on the enhanced basal collagen contraction activity of disease FPCL cultures.

## Methods

### Patient samples and primary cell cultures

Our study protocol was cleared through the UWO Ethics Committee for Research Involving Human Subjects. Areas of diseased fascia and uninvolved normal (control) PF tissue were collected during surgery. DD explant cultures were initially cultured in starter media consisting of α-MEM (Gibco, Invitrogen Corporation) supplemented with 20% fetal bovine serum (FBS, Clontech Laboratories, Palo Alto, CA), and antibiotics (Penicillin G and streptomycin sulfate) and fungizone (Gibco, Invitrogen Corporation) as previously described [23]. Established primary culture lines were maintained in α-MEM + 10% FBS + antibiotics + fungizone. Culture flasks were incubated at 37°C in a humidified chamber with 5% CO_2_. Medium was changed every 4–5 days and the cells sub-cultured using 0.05% Trypsin-EDTA (Gibco, Invitrogen Corporation, Grand Island, NY) when confluent.

### Fibroblast Populated Collagen Lattice (FPCL) contraction assay

Collagen contraction was carried out using patient-matched disease (D) and control (C) primary cultures (passages 2 – 6) established from patient-matched DD lesions and uninvolved palmar fascia (control). Collagen lattices were prepared by mixing cell suspensions with a neutralized solution of collagen type I matrix (8 parts Vitrogen100 collagen type I, 2.9 mg/ml, Collagen Corp, Santa Clara, CA, USA + 1 part 10 × α-MEM + 1 part HEPES buffer, pH 9.0). The cell-collagen concentrations were adjusted with sterile phosphate buffered solution (PBS) to attain a final collagen concentration of 2.0 mg/ml and a final cell concentration of 8.6 × 10^4 ^cells/ml of matrix. The cell-collagen mixture was then aliquoted into 24 well culture dishes (0.5 ml/well) that were pre-treated with a PBS solution containing 2% (w/v) bovine serum albumin (BSA). Following FPCL polymerization (1 hr, 37°C) culture medium (0.5 ml) consisting of α-MEM + 10% fetal bovine serum (FBS) was added atop each lattice. After 2 days of culture the attached FPCLs were mechanically released from the sides of the culture plates. Digital images of the contracting FPCL were captured at various time points over a 5-day assay period using a conventional flatbed scanner. Collagen lattice areas were then quantified using the Image J program [31]. Each assay was done in quadruplicate.

### TGF-β_2 _and neutralizing anti-TGF-β_2 _antibody treatments

Commercially available human recombinant TGF-β_2 _(expressed in NSO murine myeloma cells) was acid activated in a solution of 4N HCl + 0.1% (w/v) BSA according to the manufacturer's instructions (Product # T 2815, Sigma, St. Louis, MO). Acid activated human recombinant TGF-β_2 _was then aliquoted and frozen for extended storage at -70°C. The indicated concentrations of activated human recombinant TGF-β_2 _were added to complete culture media immediately following FPCL polymerization. Neutralizing anti-TGF-β_2 _antibodies (R&D Systems, Minneapolis, MN) were added to complete culture media either immediately following FPCL polymerization or added to the cell suspension prior to mixing with the neutralized Vitrogen collagen type I solution. Control FPCL cultures were treated with appropriate carrier solutions.

### Cell proliferation assay

FPCL cultures were assayed using a commercially available CellTiter 96^® ^A_queous _One Solution cell proliferation assay according to the manufacturer's instructions (Promega, Madison, WI, USA). This cell proliferation assay is a colorimetric method that uses a tetrazolium compound 3-(4,5-dimethylthiazol-2-yl)-5-(3-carboxymethoxyphenyl)-2-(4-sulfophenyl)-2H-tetrazolium salt (MTS) in combination with a stable electron coupling reagent PES (phenazine ethosulfate). This produces a chemically stable MTS tetrazolium compound that can be bio-reduced by cells to form a soluble colored formazan product [32,33]. Briefly, cells from patient-matched primary cultures were cultured as stressed-relaxed FPCLs as described above. Each primary cell line was plated as quadruplicate FPCL cultures for each of the indicated time points with 0.5 ml of culture media ± TGF-β_2 _(1 ng/ml) atop each FPCL. At each of the designated contracting time points 100μl of CellTiter 96^® ^A_queous _One solution reagent was added to the FPCL cultures and incubated for 3 hours (37°C, 5% CO_2 _atmosphere, humidified chamber). FPCL media was then collected and aliquoted into a 96-well culture plate for absorbance reading at 450 and 650 nm (background reference λ) using a 96-well BIO-RAD microplate reader. Media was also collected from FPCLs containing no cells (negative control) for background absorbance readings. The assay was conducted over 5 days during the course of FPCL contraction. A standard curve was also generated to calculate relative cell numbers per FPCL (range of 4 × 10^3^–10^5 ^cells/FPCL).

### Statistical analysis

Student two-tailed *t *test was used to compare data between two groups. Values were expressed as mean ± standard deviation of the mean (SDM). *P *values < 0.05 were considered statistically significant.

## Results

### TGF-β_2 _stimulates FPCL contraction

**Figure 1:**
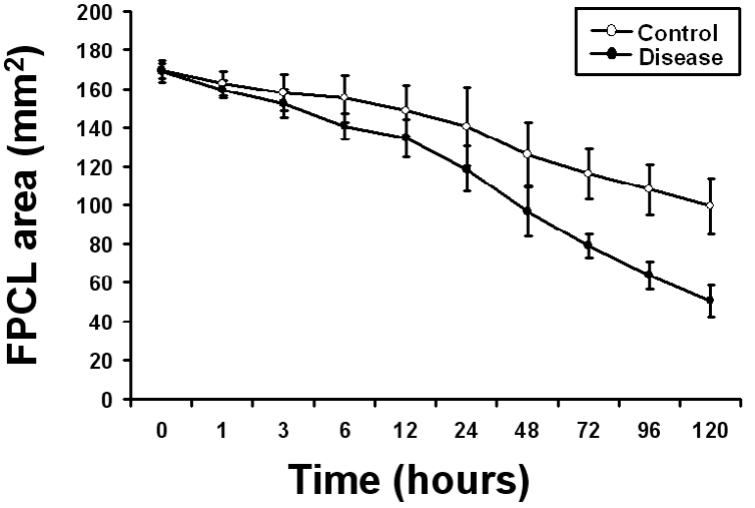
**Collagen contraction of primary control and disease FPCL cultures**. Digital images of contracting FPCL cultures were captured and analyzed using Image J software to quantify collagen contraction. The mean FPCL surface area (mm^2^) was plotted over time with each data point representing the mean ± the standard deviation of the mean (SDM) of three independent patient-matched cell lines. Experiments were repeated in quadruplicate.

**Figure 2:**
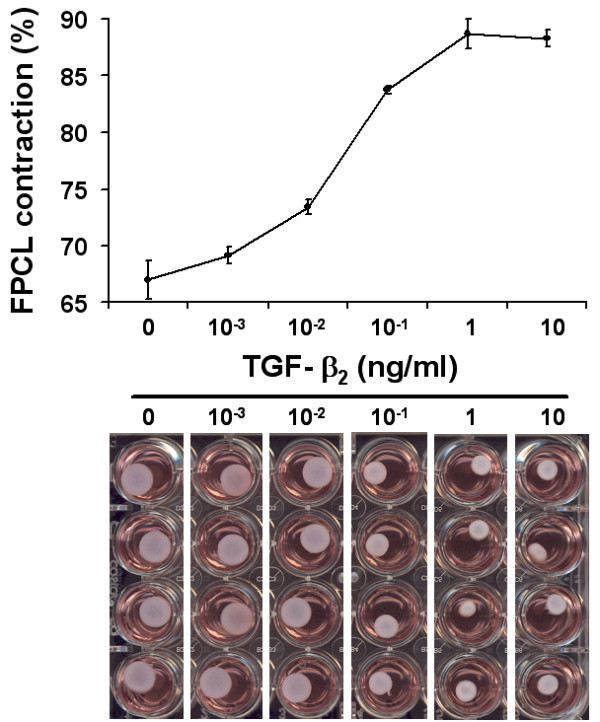
**TGF-β_2 _dose response**. The dose response curve (**upper panel**) shows the accumulated FPCL contraction (%) over 5 days. The degree of TGF-β_2 _stimulated contraction was measured over a large concentration range (>5 orders of magnitude). The plotted data shows a typical dose-response relationship, with maximal response elicited by 1 ng/ml of TGF-β_2_. Representative images of contracting FPCL after 5 days are shown for increasing concentrations of TGF-β_2 _(**lower panel**).

**Figure 3:**
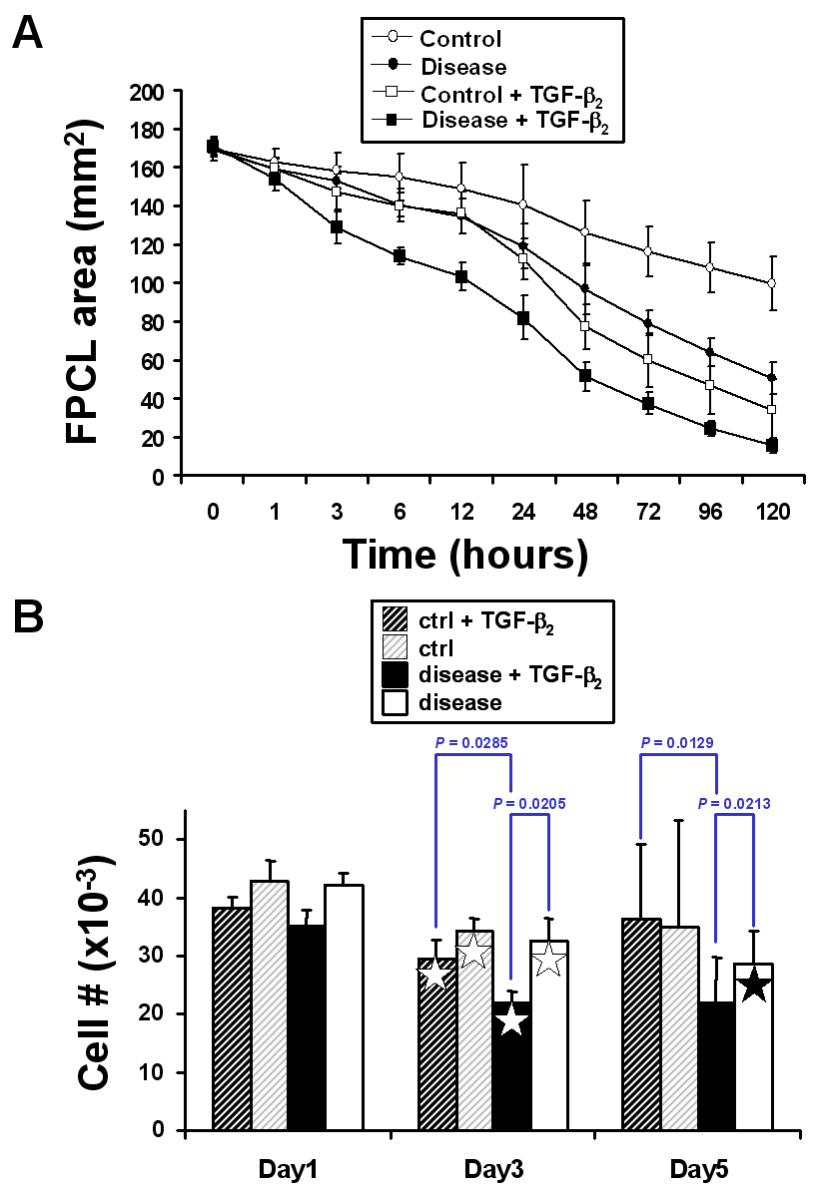
**FPCL contraction in response to exogenous TGF-β_2_**. Contracting FPCL cultures (± 1 ng/ml TGF-β_2_) were analyzed using Image J software to quantify collagen contraction. (**A**) The plotted data points represent the mean surface area ± SDM for three independent patient-matched primary FPCL cultures. Experiments were repeated in quadruplicate. (**B**) Cell proliferation/viability assays were performed on contracting FPCL cultures. The plotted bar graph represents the mean cell number ± SDM for the indicated time points for one representative patient-matched disease and control primary culture. Significant differences between groups are indicated by the *P*-values. The stars denote significance differences (*P *< 0.05) between the same treatment groups of different time points (white star – Day 1 vs. Day 3, black star – Day 1 vs. Day 5).

We first examined the basal collagen contraction activity of three independent patient-matched early passage control and disease primary FPCL cultures. As shown in Figure 1, disease FPCL cultures contracted collagen faster and to a greater degree when compared to patient-matched control FPCL cultures. This is in agreement with previous results that show distinct phenotypic differences between control and disease primary DD cultures [22,24], including enhanced collagen contraction by disease FPCL cultures [23]. Next, we explored the role of exogenous TGF-β_2 _on FPCL contraction. Initial experiments showed a typical dose-dependent response for TGF-β_2 _stimulated FPCL contraction (Fig. 2). Because maximum collagen contraction was achieved in response to 1 ng/ml of TGF-β_2_, all subsequent experiments used this dose. As shown in Figure 3a, we observed enhanced contraction rates and total collagen contraction for both control and disease cells treated with exogenous (1 ng/ml) TGF-β_2_. This enhanced collagen contraction activity observed in disease FPCL cultures was not due to differences in cell proliferation/viability between control or disease FPCL cultures. In fact, TGF-β_2 _appears to exert a significant pro-apoptotic effect on relaxed disease FPCL cultures that is absent in the 'control' FPCL cultures (Fig 3b). Thus, the amount of collagen contraction exerted by disease FPCL cultures is more pronounced if one also considers the changes in cell viability over the course of FPCL contraction.

### Neutralizing anti-TGF-β_2 _antibodies block TGF-β_2 _stimulated FPCL contraction but do not alter basal FPCL contraction

**Figure 4:**
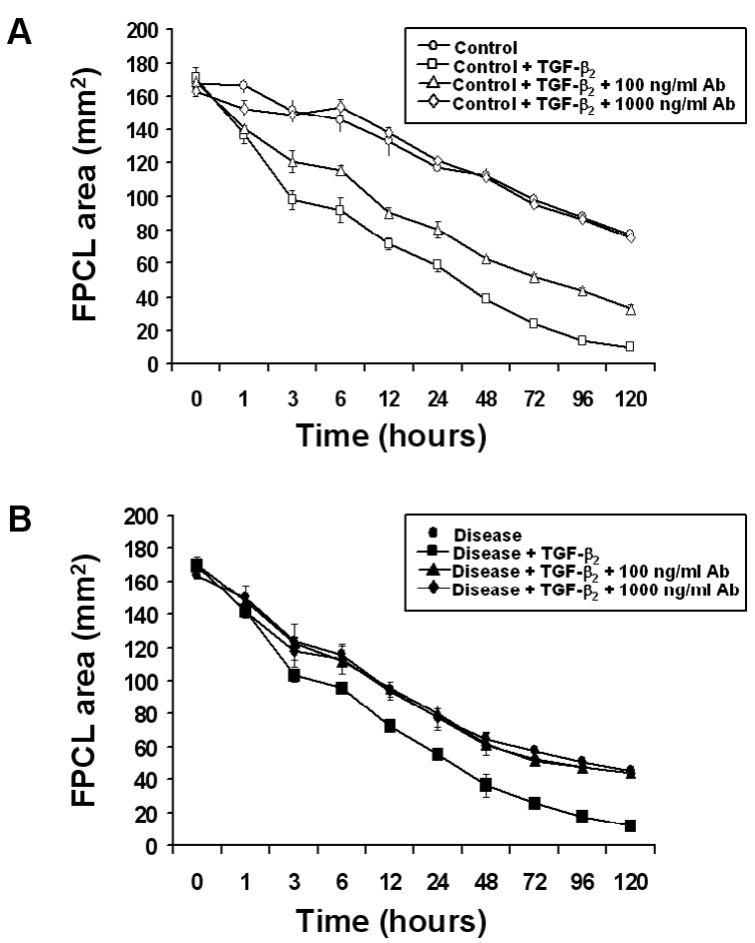
**Neutralizing anti-TGF-β_2 _antibodies block TGF-β_2 _stimulated FPCL contraction**. (**A**) Control and (**B**) disease FPCL cultures (± 1 ng/ml TGF-β_2_) were treated with the indicated concentrations of neutralizing anti-TGF-β_2 _antibodies. FPCL contraction was analyzed and plotted as the mean surface area ± SDM for quadruplicate cultures per treatment.

**Figure 5:**
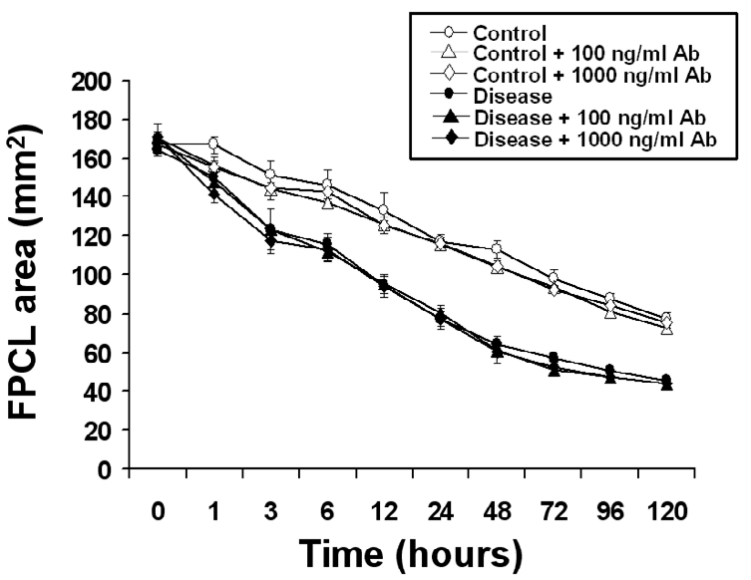
**Neutralizing anti-TGF-β_2 _antibodies do not inhibit basal collagen contraction activity of control or disease FPCL cultures**. Disease and control FPCL cultures were incubated in the absence or presence of the indicated concentrations of neutralizing anti-TGF-β_2 _antibodies. Measurements of the contracting FPCL were plotted as the mean surface area ± SDM for quadruplicate cultures per treatment.

Exogenous TGF-β_2 _stimulated collagen contraction in both control and disease FPCL cultures was inhibited in a dose-dependent manner by neutralizing anti-TGF-β_2 _antibodies. As shown in Figure 4, lower concentrations of neutralizing antibodies (100 ng/ml) partially inhibited FPCL contraction, while higher concentrations of neutralizing antibodies (1000 ng/ml) completely inhibited TGF-β_2 _stimulated FPCL contraction, thus confirming the ligand-dependent nature of this response *in vitro*. We also examined the effect of neutralizing anti-TGF-β_2 _antibodies on the basal collagen contraction activity of primary disease and control FPCL cultures. As shown in Figure 5, high concentrations (1000 ng/ml) of neutralizing anti-TGF-β_2 _antibodies had no effect on the basal levels of collagen contraction observed in either control or disease FPCL cultures, suggesting that there is little or no endogenous active TGF-β_2 _that could account for the FPCL contraction *in vitro*. To preclude that more localized interactions between endogenously produced TGF-β_2 _and its cell-surface receptors may account for the observed increase in disease FPCL contraction, we also pre-incubated the cells with neutralizing anti-TGF-β_2 _antibodies prior to forming FPCL. Regardless, the blocking antibodies had no effect on the basal levels of FPCL contraction (data not shown).

## Discussion

Members of the TGF-β family are potent fibrogenic factors that play an important role in the patho-physiology of numerous fibro-proliferative disorders, including DD [34]. The study presented here focused on the effect of TGF-β_2 _on collagen contraction using primary FPCL cultures derived from patient-matched disease and control unaffected PF tissue. Here, we found that disease FPCL cultures contracted collagen faster and to a greater degree than patient-matched control FPCL cultures. Although neutralizing anti-TGF-β_2 _antibodies effectively blocked exogenous TGF-β_2 _stimulated FPCL contraction for both control and disease cultures, the same neutralizing antibodies had no effect on the basal collagen contraction activity of either disease or control FPCL cultures, suggesting that enhanced disease FPCL contraction is not due to elevated levels of endogenous active TGF-β_2_. These results, however, do not exclude the possibility that there may be differences in the levels of latent TGF-β_2 _produced by these cell cultures that may be subsequently activated *in vivo*. Recent work by Kuhn et. al. (2002) reported reduced DD FPCL contraction in response to tamoxifen, which was associated with a decreased TGF-β_2 _production [35]. However, the TGF-β_2 _assayed in this study required an acid-activation step, suggesting that TGF-β_2 _produced by these cells is largely in its latent, non-activate form. Thus, it is possible that TGF-β_2 _produced by DD fibroblasts and/or other resident cell types *in vivo *may be important to disease cord contraction, provided appropriate regulators of latent TGF-β activation are also present and active. However, it does not explain the enhanced basal FPCL contraction rates of disease cell cultures, suggesting that other signalling factors may be regulating this disease cell function *in vitro*. It is interesting to note that both TGF-β and β-catenin signalling pathways show some degree of 'cross-talk' [29,30,36-38], suggesting that β-catenin plays an important role in TGF-β signalling. The degree and significance of signalling 'cross-talk' between β-catenin and TGF-β in the context of DD is unknown and needs further examination.

Although previous DD studies have used different 'control' primary fibroblast as 'disease-free' controls, namely fibroblasts derived from transverse carpal ligament material obtained from patients undergoing carpal tunnel release (CTR), they have the disadvantage of being of different anatomical origin than PF derived 'control' fibroblast cultures. This is an important consideration given that fibroblasts exhibit functional heterogeneity depending on their origin [39-41]. For example, human fibroblasts that express the cell surface antigen Thy-1 are capable of TGF-β_1 _stimulated myofibroblast differentiation, while Thy-1 negative fibroblasts appear to be only capable of lipofibroblast differentiation [42]. Phenotypic differences have also been attributed to fibroblasts of different dermal origins [43]. For example, Chipev and colleagues showed that TGF-β_1 _had a pro-apoptotic effect on non-palmoplantar (keloid) fibroblasts and an anti-apoptotic effect on palmoplantar fibroblasts. Similar to what we have observed for detached or contracting DD FPCL cultures, they showed that TGF-β_1 _treated (in the presence of serum) keloid FPCL cultures underwent the most extensive apoptosis response upon mechanical release compared to dermal fibroblasts from different body sites [43]. Perhaps the similar myofibroblast phenotype attributed to both DD and keloid fibroblast cultures also dictates a similar apoptotic fate to relaxed FPCL cultures in response to TGF-βs. Although the loss of tension in these cultures triggered a mostly uniform loss in cell viability across all groups, unlike disease FPCL cultures TGF-β_2 _did not appear to have any significant pro-apoptotic effect on relaxed 'control' FPCL cultures. In light of these and other findings, it appears that the patient-matched control fibroblast cultures employed in these studies may truly represent a suitable 'control' phenotype, with the added advantage of having the same PF origins as the disease cell cultures. While this does not exclude the possibility that the control cultures may harbour some residual disease cells, this is not supported by the distinct phenotypic differences we have observed between these two types of patient-matched PF cultures in the current and previous studies. In these earlier studies, we reported elevated levels of β-catenin and fibronectin isoforms in disease FPCL cultures [22-24], as well as enhanced disease FPCL contraction rates which we have subsequently confirmed in these studies. This together with the distinctive pro-apoptotic affects of TGF-β_2 _on disease FPCL cultures described here, further support the notion that the patient/tissue-matched 'control' cultures have a non-disease phenotype that is suitable for these types of investigations.

Although the 'synthetic' myofibroblast features of the disease cells described in previous studies are known to be stimulated by TGF-β [20,21,25-30,35], our results suggests that endogenous TGF-β_2 _does not play a role in regulating these phenotypic differences *in vitro*. Nevertheless, aberrant expression of various TGF-β signalling components have been previously shown to trigger this 'synthetic' myofibroblast phenotype in other fibro-proliferative disorders, specifically keloids and burn hypertrophic scarring [34,44,45], that can to some extent be inhibited by neutralizing anti-TGF-β_2 _antibodies [46,47]. Hopefully future studies will unravel the extent of these phenotypic differences between these patient/tissue-matched control and disease FPCL cultures with respect to pro-fibrotic factors like TGF-β_2_, tension and other important intersecting signaling pathways.

## Conclusions

Primary disease FPCL cultures contract collagen faster and to a greater extent than control PF-matched FPCL cultures. While neutralizing anti-TGF-β_2 _antibodies can block exogenous TGF-β_2 _stimulated collagen contraction for both control and disease FPCL cultures, it had no effect on the basal contraction rates of either control or disease FPCL cultures. We, therefore, conclude that the enhanced collagen contraction activity of disease FPCL cultures is not due to differences in the levels of endogenous active TGF-β_2_.

## List of abbreviations

DD, Dupuytren's disease; PF, palmar fascia; CTR, carpal tunnel release; TGF-β, transforming growth factor-beta; FPCL, fibroblast populated collagen matrix; PBS, phosphate buffered saline; RT, room temperature; HEPES, 4-(2-hydroxyethyl)-1-piperazineethanesulfonic acid; α-MEM, alpha-minimal essential medium; FBS, fetal bovine serum; Fn, fibronectin; MTS, 3-(4,5-dimethylthiazol-2-yl)-5-(3-carboxymethoxyphenyl)-2-(4-sulfophenyl)-2H-tetrazolium salt; PES, phenazine ethosulfate; RT-PCR, reverse transcriptase polymerase chain reaction; SDM, standard deviation of the mean.

## Competing interests

The authors declare that they have no competing interests.

## Authors' contributions

RT and YW carried out the experiments. RT drafted the manuscript. JCH and BSG designed and coordinated the research, and reviewed and edited the manuscript. All authors read and approved the final manuscript.

## Pre-publication history

The pre-publication history for this paper can be accessed here:
